# The Complete Genome Sequence of *Conuropsis carolinensis*, the Carolina Parakeet

**DOI:** 10.56179/001c.66227

**Published:** 2022-12-30

**Authors:** Taylor Hains, Stacy Pirro, John M Bates, Shannon J Hackett

**Affiliations:** 1 Negaunee Integrative Research Center, Field Museum of Natural History,; 2 Committee on Evolutionary Biology, University of Chicago,; 3 Biodiversity, Iridian Genomes

**Keywords:** genome, bird, parrot, extinct

## Abstract

The Carolina Parakeet (*Conuropsis carolinensis*) is an extinct species of parrot that was native to the eastern, midwest, and plains regions of the United States. We present the whole genome sequence of this species. Illumina sequencing was performed on a genetic sample from a single captive individual. The reads were assembled using a de novo method followed by a series of references from related species for finishing. The raw and assembled data is publicly available via Genbank: Sequence Read Archive (SRR21023482) and assembled genome (JAOBYI000000000).

## Introduction

The Carolina Parakeet was a small green parakeet neotropical parrot with a bright yellow head, reddish orange face and pale beak. It lived in old-growth forests along rivers and in swamps in the eastern, midwest, and plains regions of the United States. Though formerly prevalent within its range, the bird had become rare by the middle of the 19th century. The last known specimen perished in captivity at the Cincinnati Zoo in 1918, and the species was declared extinct in 1939 (Burgio, Carlson, and Tingley 2017).

## Methods

The genetic sample was provided by the Field Museum of Natural History, catalogue number FMNH 124024.

DNA extraction was performed using the Qiagen DNAeasy genomic extraction kit using the standard process. A paired-end sequencing library was constructed using the Illumina TruSeq kit according to the manufacturer’s instructions. The library was sequenced on an Illumina Hi-Seq platform in paired-end, 2 × 150bp format. The resulting fastq files were trimmed of adapter/primer sequence and low-quality regions with Trimmomatic v0.33 (Bolger, Lohse, and Usadel 2014). The trimmed sequence was assembled by SPAdes v2.5 (Bankevich et al. 2012) followed by a finishing step using Zanfona (Kieras, O’Neill, and Pirro 2021).

## Results and Data Availability

Raw and assembled data is publicly available via GenBank:

### Raw genome data

https://trace.ncbi.nlm.nih.gov/Traces/sra/?run=SRR21023482

### Assembled genome

https://www.ncbi.nlm.nih.gov/nuccore/JAOBYI000000000
